# *Helicobacter pylori* Outer Membrane Protein-Related Pathogenesis

**DOI:** 10.3390/toxins9030101

**Published:** 2017-03-11

**Authors:** Yuichi Matsuo, Yasutoshi Kido, Yoshio Yamaoka

**Affiliations:** 1 Department of Environmental and Preventive Medicine, Oita University Faculty of Medicine, 1-1 Idaigaoka, Hasama-Machi, Yufu-City, Oita 879-5593, Japan; m13d9013@oita-u.ac.jp (Y.M.); kidoyasu@oita-u.ac.jp (Y.K.); 2 Department of Medicine-Gastroenterology, Michael E. DeBakey Veterans Affairs Medical Center and Baylor College of Medicine, Houston, TX 77030, USA

**Keywords:** *Helicobacter pylori*, outer membrane protein, pathogenesis, Type IV secretion system

## Abstract

*Helicobacter pylori* colonizes the human stomach and induces inflammation, and in some cases persistent infection can result in gastric cancer. Attachment to the gastric mucosa is the first step in establishing bacterial colonization, and outer membrane proteins (OMPs) play a pivotal role in binding to human cells. Some OMP interaction molecules are known in *H. pylori*, and their associated host cell responses have been gradually clarified. Many studies have demonstrated that OMPs are essential to CagA translocation into gastric cells via the Type IV secretion system of *H. pylori*. This review summarizes the mechanisms through which *H. pylori* utilizes OMPs to colonize the human stomach and how OMPs cooperate with the Type IV secretion system.

## 1. Introduction

Membrane proteins are classified by configuration, which determines their function and location. α-Helical membrane proteins are found in almost all membranes; however, β-barrel membrane proteins are only found in the outer membranes of Gram-negative bacteria, and not in Gram-positive bacteria. In bacteria, β-barrel outer membrane proteins (OMPs) have many functions including importing nutrients and translating signals from the outside environment. With respect to human infection, OMPs play a pivotal role in colonization, the expression of pathogenic factors, and antibiotic resistance. For *Acinetobacter baumannii*, OMPs are essential for establishing infection, and an interaction network with β-lactamase regulates antibiotic inactivation [1,2]. Thus, the role of OMPs in bacteria has been clarified, and new mechanisms to understand the biological roles of OMPs have been provided. For *Helicobacter pylori*, many virulence factors have been identified, and their functions have been gradually revealed. In addition, interaction partners of OMPs have been identified and their biological functions were elucidated. Recently, the interplay between OMPs and virulence factors has been suggested. However, the dynamic functions of OMPs in *H. pylori* are still unknown, and investigating these aspects represents an exciting field.

*H. pylori* is a Gram-negative bacterium, with which half of the world’s population is infected. This species is a Class I carcinogen that colonizes the human stomach, wherein it can induce inflammatory disorders (chronic gastritis and ulceration) and malignant neoplastic diseases (mucosa-associated lymphoid tissue and gastric cancer). *H. pylori* has mechanisms to survive the human stomach, which is a very harsh environment in which it is difficult for bacteria to survive. As a first line of defense, urease is produced by *H. pylori*, which results in the generation of ammonia to neutralize the acidic condition. Subsequently, *H. pylori* attaches to gastric epithelial cells via OMPs. The outer membrane of *H. pylori* plays a pivotal role in the attachment and colonization of gastric cells. After colonization, *H. pylori* pathogenesis results in inflammation. *H. pylori*-infected patients exhibit inflammation of the gastric mucosa, which can result in metaplasia. Finally, some *H. pylori*-infected patients develop gastric cancer; however, this typically requires long-term, persistent infection.

Among the virulence factors of *H. pylori*, cytotoxin-associated gene A product (CagA), urease, γ-glutamine transferase, high temperature requirement A, and vacuolation associated gene A (VacA) were identified as secreted [3]. Specifically, CagA is injected into human gastric cells via the Type IV secretion system (T4SS) of *H. pylori* to induce the production of inflammatory cytokines in gastric epithelial cells [4,5,6]. Recently, many studies have suggested that the OMPs of *H. pylori* play a role not only in the attachment to gastric cells but also in the enhancement of CagA translocation via the T4SS. In 1997, the genome of *H. pylori* strain 26695 was sequenced completely, and it was suggested to encode 32 OMPs [7]. Subsequent studies revealed that sialic acid-binding adhesin (SabA), blood-group-antigen-binding adhesin (BabA), adherence-associated lipoprotein A and B (AlpA/B), outer inflammatory protein A (OipA), and *Helicobacter* outer membrane protein Q (HopQ) were involved in adhesion (Table 1). Therefore, OMPs are important for colonization and the establishment of inflammation in the stomach. The functions of OMPs are summarized in this review to provide an overview of how *H. pylori* utilizes membrane proteins to colonize the stomach, a process that results in pathogenesis. Additionally, the evidence suggesting that OMPs cooperate with the T4SS to translocate CagA into gastric cells is summarized. The biological function of CagA protein has been revealed; however, how OMPs cooperate with Type IV secretion system is unclear. Thus, this review contributes to a deeper understanding of the dynamic action between OMP and CagA translocation via the T4SS. 

## 2. The Genetic Features of Helicobacter OMPs

Members of the large *Helicobacter* outer membrane protein (Hop) family were first characterized as OMPs in *H. pylori*. Five proteins (HopA–E) were isolated and these were demonstrated to function as porins for transmitting small hydrophobic molecules, nutrients, and some antibiotics by passive diffusion [8,9]. The genome of *H. pylori* 26695 was completely sequenced, and subsequent analysis identified 21 members of the Hop family; these genes encoded proteins that possess one domain of similarity at the N-terminal end and seven domains of similarity at their C-terminal ends [7]. A C-terminal phenylalanine residue is essential for the correct assembly of bacterial OMPs [10,11]. However, half of the Hop family proteins contain a C-terminal tyrosine. In addition, all Hop proteins possess highly similar C-termini, which contain aromatic residues. With phenylalanine at their C-terminal ends, Hop proteins are less divergent in this region, and thus the central hyper-variable domain contains the majority of the membrane-specific sequence. Indeed, for several Hop proteins with phenylalanine at the C-terminal end, the similarity of this region between paralogues within a strain is higher than that of orthologous proteins between isolates. *H. pylori* also contains orthologues of hop-related (Hor) protein, *Helicobacter* OMP family (Hof) protein, and *Helicobacter* outer membrane (Hom) family proteins. The functions of these proteins have not been revealed.

Most Hop proteins are predicted to fold into anti-parallel amphipathic β-sheets that organize into a β-barrel structure. Typically, β-sheet domains localize to the membrane region, and these domains are connected by short amino acid loops in the periplasmic region, whereas longer amino acid loops connect these domains in the extracellular region. It is known that the membrane domain is difficult to overexpress and purify. For *H. pylori*, the extracellular domain of OMP was recently purified and crystallized. However, the full length OMP has not yet been purified for *H. pylori*.

## 3. AlpA/B

AlpA and AlpB are involved in the adhesion of the pathogen to gastric epithelial cells [12]. Collagen IV and laminin, which exist in the extracellular matrix, are the suggested binding partners of BabA and BabB [13,14]. Both genes have high homology, specifically 46.7% in *H. pylori* 26695, and both proteins are important for adhesion to gastric cells [7]. In addition, Odenbreit et al. reported that *H. pylori* expresses AlpA and AlpB; however, all other OMPs (BabA, BabB, BabC, HopM, SabA, and OipA) were shown to be produced depending on clinical isolate strains [15]. This indicates that AlpA and AlpB are essential and play a main role in colonization. Subsequently, studies demonstrated that AlpA- and AlpB-mutant *H. pylori* strains poorly colonize the stomachs of guinea pigs, mice, and Mongolian gerbils [14,16,17]. Thus, it is clear that these factors are important for bacterial colonization. However, the related role in pathogenesis of AlpA and AlpB is controversial. Lu et al. suggested that these proteins do not affect CagA translocation via the T4SS and that they are involved in the stimulation of certain signaling pathways (MAPKs, c-Fos, c-Jun, CREB, AP-1, and NF-κB pathway), which can induce production of interleukin-8 (IL-8). Furthermore, these factors are associated with a reduction in chemokine KC and interleukin-6 in the gastric mucosa [18]. In contrast, AlpA- and AlpB-mutant *H. pylori* caused a more severe inflammatory reaction in Mongolian gerbils than the wild type [16]. The reason for the contradictory results might be due to differences in host animal species or *H. pylori* strains.

## 4. SabA

*H. pylori* adhesion via SabA is important for colonization and the induction of inflammation in the gastric mucosa. Sialyl LewisX/a glycosphingolipid (sLe^x^ and sLe^a^) was identified as a receptor for a 70 kDa protein in *H. pylori*, and this was named SabA [18,19,20]. Recent reports revealed the three-dimensional structure of the extracellular domain of SabA and identified the domain responsible for binding to sLe^x^ and LewisX antigen [21]. The functional status of SabA is regulated by slipped strand mispairing, which is determined by the number of CT dinucleotide repeats in the 5′ region of the gene. Switch “on” means that SabA is functional, whereas Switch “off” means that it is non-functional [7,22,23].

**Figure 1:**
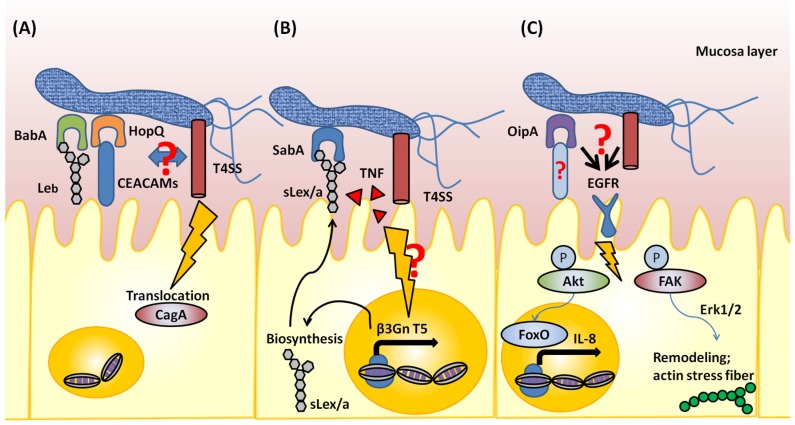
Schematic of outer membrane protein-mediated pathogenesis. (**A**) BabA interacts with Le^a^ antigen and enhances CagA translocation via the Type 4 secretion system (T4SS). HopQ interacts with CEACAMs, and is essential for CagA translocation. BabA and HopQ might interact with the T4SS in the membrane region of *H. pylori*, although this is unclear. (**B**) Absence of sLe^x^ antigen expression in the healthy stomach. *H. pylori* infection induces β3GnT5 expression in gastric epithelial cells and biosynthesis of the sLe^x^ antigen; sLe^x^ localizes to the membrane region of gastric epithelial cells. As a result, *H. pylori* can colonize by utilizing SabA, which interacts with the sLe^x^ antigen. Although the detailed mechanism is unclear, TNF and the T4SS are suggested to induce β3GnT5 expression. (**C**) OipA is suggested to induce phosphorylation of EGFR, leading to activation of focal adhesion kinase (FAK) and Akt-related signaling. Activated FAK induces actin stress fiber remodeling via the MAPK and Erk1/2 signaling pathway. In addition, phosphorylation of Akt can activate FoxO transcription factors and induce IL-8 production. The binding partner of OipA and whether OipA can cooperate with the T4SS are unclear.

The adhesion process via SabA is dynamic and co-operates with the T4SS and TNF. In fact, sLe^x^ antigen is absent in healthy gastric mucosa, and it was previously unclear how *H. pylori* uses sLe^x^ antigen for adhesion. The T4SS and TNF is suggested to induce sLe^x^ expression. Marcos et al. demonstrated that *cag PAI*-positive *H. pylori* induces β3 GlcNAcT5 (β3GnT5) expression in gastric epithelial cells. β3GnT5 is a glycosyltransferase and is essential for the biosynthesis of the Lewis antigens. Furthermore, the induction of β3GnT5 is dependent on TNF, but not on IL-8. In regard to the binding capacity of *H. pylori* via SabA, β3GnT5 expression was shown to increase the cell adhesion rates for SabA-positive *H. pylori* strains [24]. In addition, acid-responsive signaling in *H. pylori* regulates SabA transcription [25]. These data indicate that *H. pylori* adaptation to the environment is a precursor of adhesion via SabA; subsequently, inflammation might support *H. pylori* colonization of gastric epithelial cells (Figure 1).

## 5. BabA

BabA was identified as the first adhesin molecule of *H. pylori*, and this protein can bind to the Lewis B (Le^b^) blood group antigen [26,27]. Subsequent studies demonstrated that BabA has adapted to fucosylated blood group antigens, which are most prevalent in certain local populations [28]. In Europe and the United States, where Blood Groups A, B, and O are common, *H. pylori* strains can bind to Blood Groups A, B, and O Type 1 determinants (H Type 1 determinants); this is referred to as a “generalist strain.” Meanwhile, a “specialist strain” can bind to H Type 1 determinants only. A specialist strain was found in the South American native population, in which only the Blood Group O phenotype is present. Recently, the Globo H hexaglycosylceramide, e.g., the Blood Group O determinant on a Type 4 core chain, was suggested to be a novel glycosphingolipid binding partner for BabA [29]. Both strains can bind the Globo H hexaglycosylceramide; however, only the generalist strain can bind the Globo A heptaglycosylceramide, e.g., the Blood Group A determinant on a Type 4 core chain.

BabA is suggested to enhance CagA translocation into gastric cells and induce severe inflammation in the stomach (Figure 1) [30]. Triple-positive (BabA, *vacA*-s1 type, CagA) *H. pylori* can colonize a greater proportion of the gastric mucosa, and induce severe inflammation in the stomach [31]. In addition, triple-positive *H. pylori*-infected patients exhibit a higher incidence of intestinal metaplasia, compared to that in patients infected with double-positive (*vacA*-s1 type, CagA) *H. pylori* [32]. Recently, the extracellular domain of BabA was crystallized, and this provided new insights into the protein features. Moonens et al. identified the domain of BabA that is responsible for interaction with blood group antigens. Furthermore, *N*-acetylcysteine (NAC), a redox-active compound, was shown to disrupt the disulfide bond of the interaction domain, inactivate the binding properties of BabA, and thus block BabA-mediated adherence to the gastric mucosa of mice [33]. In addition, treatment of *H. pylori*-infected Le^b^-expressing mice with NAC lowers gastric mucosal neutrophil infiltration. This might provide the basis for possible *H. pylori* eradication therapies.

## 6. OipA

OipA was initially identified as a protein that induces a pro-inflammatory response based on the fact that an *oipA* mutant resulted in the reduced production of IL-8 in a gastric epithelial cell line [34]. In contrast, some studies have indicated that OipA plays a role in *H. pylori* adhesion to host cells and does not influence the production of IL-8 in gastric cells [35,36]. Whether OipA can induce IL-8 production is controversial. The functional status of OipA is regulated by slipped strand mispairing, which is the same mechanism utilized by SabA [34,35,36,37]. Many OMP family genes demonstrate a loss of one resident locus (*hopM/N*, *babA/B/C*, and *sabA/B*), whereas it was reported that the OipA locus was duplicated, referred to as OipA-2 [38]. OipA-2 is found in *H. pylori* hspEAsia and hspAmerind strains, but not in hpEurope or hspWAfrica strains, based on multi-locus sequence typing analysis.

The role of OipA as an adhesion molecule is suggested by in vitro experiments using gastric epithelial cells [34,35,39]. However, results in an animal model indicate that the adhesive action of OipA is dependent on the *H. pylori* strain. Akanuma and colleagues reported that the *oipA*-mutant *H. pylori* TN2 strain does not infect Mongolian gerbils [40]. In contrast, Franco and co-workers reported that the *oipA*-mutant *H. pylori* 7.13 strain infected Mongolian gerbils; however, *oipA* mutant strain-infected animals did not develop gastric cancer, whereas 27% of those infected with the wild-type strain did develop this condition [41]. Thus, the adhesion of OipA depends on *H. pylori* strain and controversial. Therefore, it is difficult to demonstrate the relationships between OipA status and molecular processes as well as clinical outcomes.

Based on epidemiological studies, functional OipA correlates with other virulence factors such as *cag PAI*, *vacA*-s1 type, and *vacA*-m1 type [35,42,43,44]. Therefore, it is difficult to demonstrate the direct function of OipA by using mutant *H. pylori* strains; however, OipA-related signaling has been reported. OipA is suggested to induce the remodeling of actin stress fibers by activating the phosphorylation of focal adhesion kinase (FAK); this leads to the activation of extracellular signal-regulated kinases 1 and 2 (Erk1/2) [45]. OipA-mediated FAK activation might be a consequence of altered epidermal growth factor receptor (EGFR) signaling. *oipA* mutant *H. pylori* could not activate EGFR-related signaling involving phosphatidylinositol 3-kinase (PI3K) [45], phosphoinositol dependent kinase-1 (PDK1), and Akt, which regulate FoxO forkhead transcription factor activity, leading to IL-8 induction [46]. Recently, EGFR/FAK signaling was shown to lead to phosphorylation of paxillin and subsequent actin remodeling (Figure 1) [47].

It is difficult to demonstrate the relationships between OipA status and clinical outcomes. However, it is clear that OipA status is associated with the presence of the other virulence factors in *H. pylori*. Therefore, OipA might cooperate with other virulence factors during the pathogenesis of *H. pylori*.

## 7. HopQ

HopQ is a paralog of the Hop family that includes BabA and SabA [48]. HopQ is classified into Type I and Type II alleles, and these alleles correlate with other virulence factors. Ping and Timothy reported that Type I HopQ alleles are significantly more common in *cagA*-positive and *vacA*-s1 type strains than in *cagA*-negative and *vacA*-s2 strains [49]. Furthermore, based on an epidemiological study, it was revealed that Type I HopQ alleles are more common in East Asian *H. pylori* strains [50]. This indicates the presence of geographic differences for the HopQ allele type, as with *vacA* and *cagA* genotypes. 

HopQ was reported as a non-*cagPAI*-encoded co-factor of the T4SS. Belogolva et al. suggested that HopQ is essential for CagA translocation, and that deletion of HopQ reduces T4SS-dependent activation of signaling (NF-κB, MAPK signaling, and IL-8 production) [51]. Furthermore, this observation was reported in human granulocytes [52]. Recently, the carcinoembryonic antigen-related cell adhesion molecule family (CEACAMs) was defined as a group of host cell receptors for HopQ. CEACAMs have isoforms, and HopQ binds the amino-terminal IgV-like domain of human CEACAM1, CEACAM3, CEACAM5, and CEACAM6 proteins. Based on the crystal structure of the extracellular domain of HopQ, a β-hairpin insertion in the extracellular 3 + 4 helix bundle domain of HopQ is important for CEACAM binding, and this determines the type of HopQ allele. A peptide derived from this domain competitively inhibits HopQ-mediated Cag translocation, similar to that observed with genetic or antibody-mediated abrogation of HopQ function [53,54]. This suggests the possibility that CagA translocation is mediated not only by the T4SS, but also by membrane proteins (Figure 1). The CEACAM-binding site of HopQ has been found to exhibit sequence diversity. Therefore, such variations might affect the translocation of CagA via the T4SS.

## 8. Conclusions

*H. pylori* expresses many different virulence factors. The function of each virulence factor has gradually been revealed. In regard to membrane proteins, the binding partners of BabA, SabA, and HopQ were identified, and subsequently BabA and HopQ were suggested to enhance CagA translocation into gastric cells via the T4SS. In addition, many epidemiological studies have demonstrated that OMPs correlate with other virulence factors and clinical outcomes. Therefore, these proteins might have a dynamic function in cooperating with the T4SS and other factors. However, less is known about the interplay between membrane proteins and the membrane region of *H. pylori*. Recently, it is clear that outer membrane vesicles (OMVs) have a unique function. OMVs are involved with periplasmic and outer membrane proteins, peptideglycanes, lipopiolysaccharides, DNA, RNA, and enzymes. OMVs are also involved in a mechanism to transfer virulence factors and maintain bacteria communities. Thus, OMPs at MOVs might play an important role in communicating with bacteria. Studies that focus on local or temporal interactions among virulence factors and the other molecules are required for a deeper understanding of the dynamic pathogenesis and communication of *H. pylori*.

Many studies demonstrated that *H. pylori* utilize OMPs to colonize the human stomach. This indicates that OMPs might be a vaccine candidate. In fact, many reports suggested that OMPs might be a candidate as a vaccine based on in vitro experiments. However, there has been no report that shows that OMPs are useful as target molecules for vaccines in animal models. An animal model is essential to demonstrate the efficacy of a vaccine. Thus, this problem needs to be overcome to produce a vaccine for *H. pylori* infection.

## Figures and Tables

**Table 1:** *Helicobacter pylori* outer membrane proteins that interact with the host cell.

Outer Membrane Protein	Interaction Partner	Suggested Protein Function
BabA (HopS)	Lewis B, Globo H hexaglycosylceramide, Globo A heptaglycosylceramide	Adhesion to host cell, enhancing translocation of CagA via the T4SS
SabA (HopP)	Sialyl Lewis X, Sialyl Lewis A, Lewis X	Adhesin to host cell
OipA (HopH)	Not known	Adhesion, induction of inflammatory cytokine production
HopQ	CEACAM1, 3, 5, 6	Adhesion to host cell, translocation of CagA via the T4SS
AlpA/B (HopC/B)	Collagen IV, Laminin	Adhesion to ECM
